# Development and validation of a clinical prediction model for osteoporosis diagnosis by lumbosacral X-ray and radiomics

**DOI:** 10.3389/fragi.2025.1476902

**Published:** 2025-07-01

**Authors:** Xiaofeng Chen, Dongling Cai, Hao Li, Weijun Guo, Qian Li, Jinjun Liang, Junxian Xie, Jincheng Liu, Zhen Xiang, Wenxuan Dong, Sihong OuYang, Zhuozheng Deng, Qipeng Wei

**Affiliations:** ^1^ Department of Orthopedics, Panyu Hospital of Chinese Medicine, Guangzhou, China; ^2^ Guangzhou University of Chinese Medicine, Guangzhou, China; ^3^ Department of Dermatology, Panyu Hospital of Chinese Medicine, Guangzhou, China

**Keywords:** osteoporosis, lumbosacral X-ray, predictive model, radiomics, nomogram

## Abstract

**Purpose:**

To develop a clinical prediction model for the diagnosis of osteoporosis using lumbosacral X-ray images through radiomics analysis.

**Methods:**

A total of 272 patients who underwent dual-energy X-ray absorptiometry (DXA) and lumbosacral X-ray examinations were categorized into two groups: (1) the training set (n = 191) and (2) the validation set (n = 81). Radiomic features were extracted using 3D Slicer software, and radiomic scores were calculated using the least absolute contraction and selection operator logistic regression, facilitating the generation of radiomic features. Subsequently, a clinical model, in conjunction with the radiomic features, was employed to develop a column-line diagram for the clinical and imaging feature prediction model. Performance evaluations for various models were conducted, encompassing recognition ability, accuracy, and clinical value, with the aim of identifying and optimizing prediction models.

**Results:**

The 12 most optimal imaging features were identified. Upon comprehensive performance analysis across different models, the clinical and radiomics model emerged as the most effective. The training set and test set area under the curves (AUCs) were 0.818 and 0.740, respectively. Additionally, the model exhibited a sensitivity and specificity of 81.6%, 80.6% and 77.5%, 73.2%, respectively.

**Conclusion:**

In this study, we developed a column-line diagram that integrates clinical and radiomics feature, presenting a novel screening tool for osteoporosis in primary hospitals. This tool aims to enhance the efficiency of osteoporosis diagnosis in primary hospitals.

## Introduction

Osteoporosis (OP) is a systemic, metabolic bone disease characterized by decreased bone mass, destruction of bone microarchitecture, heightened bone fragility, diminished bone strength, and an increased risk of fractures (Svedbom et al., 2014). China has identified osteoporosis as one of the three major diseases for research concerning the elderly (Yan et al., 2016). The condition poses a significant public health challenge in the face of an aging society. In 2018, the Health Commission highlighted the limited public awareness of osteoporosis, revealing that while the prevalence among individuals over 50 years of age is 19.2%, only 3.7% of this population undergoes bone density test (Epidemiological Survey on Osteoporosis in, 2019). Moreover, fragility fractures resulting from osteoporosis have catastrophic implications for the health and quality of life of middle-aged and elderly individuals. It is anticipated that by 2025, there will be approximately 5.99 million osteoporosis-related fractures (Si et al., 2015), imposing a considerable healthcare cost estimated at $25.43 billion and significantly compromising the quality of life for the aging population.

Presently, the awareness rate among patients over 50 years old is only 7.0%. For women in the same group, the proportion who have undergone a bone density test is only 4.3%. Moreover, the diagnosis rate of osteoporosis among individuals who have experienced a fragility fracture is only two-thirds, and the utilization of effective anti-osteoporosis medication remains below 25% (Wang et al., 2015). The diagnosis of osteoporosis relies on measuring bone mineral density through dual-energy X-ray absorptiometry (DXA). However, the majority of hospitals and communities lack the capability to conduct osteoporosis diagnoses, thereby impeding early detection and treatment efforts. Therefore, there is an urgent need to establish a multidimensional, universally applicable screening method for osteoporosis that can be implemented across all levels of healthcare organizations.

In 2012, Dutch scholars, La et al. (2012), pioneered the concept of imaging genomics, emphasizing the utilization of computer software to extract quantitative features from medical images. Leveraging big data analytics, this approach facilitates the analysis of clinical information to guide clinical decision-making. Imaging genomics has already demonstrated a significant role in tumor research and various other fields (Song et al., 2018; Hu et al., 2019; Huang et al., 2016). While some scholars have previously explored the diagnosis of osteoporosis by radiomics feature through computed tomography (CT) and magnetic resonance imaging (MRI), achieving a certain degree of accuracy (Huang et al., 2022), certain primary hospitals that lack access to CT and MRI technologies face limitations in utilizing these methods for the diagnosis of osteoporosis.In clinical practice in China, lumbosacral X-rays are commonly performed for patients with low back pain or suspected degenerative conditions. In comparison to CT and MRI, lumbosacral X-ray offers advantages such as cost-effectiveness, lower radiation exposure, and broader accessibility. Therefore, this study developed and validated a clinical prediction model for osteoporosis diagnosis through lumbosacral X-ray, serving as a valuable tool for the primary screening of osteoporosis.

## Materials and methods

### Study participants

**FIGURE 1:**
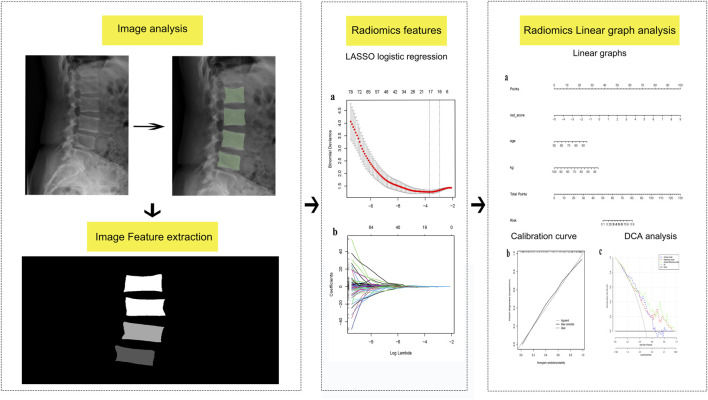
Flow chart of the study.

Data from patients aged 50 years or older were retrospectively collected from the orthopedic department of Guangzhou Panyu District Hospital of Traditional Chinese Medicine between June 2020 and June 2022. The inclusion criteria encompassed the following: (1) individuals who underwent lumbosacral X-ray and DXA, and (2) the time interval between the two examinations being less than 3 months; (3) those aged ≥50 years. The exclusion criteria were as follows: (1) individuals missing DXA or lumbosacral X-ray; (2) a prior history of lumbosacral surgery with internal fixation; (3) Patients with L1-L4 vertebral fractures; (4) a history of major internal medicine-related diseases, such as hyperthyroidism, hypocalcemia, and tumors. The diagnosis of osteoporosis was determined based on the European Clinical Guidelines on Osteoporosis (Kanis et al., 2013), with osteoporosis defined as T ≤ −2.5 and non-osteoporosis as T > −2.5. A total of 272 cases met the inclusion criteria and were categorized into osteoporosis and non-osteoporosis groups based on T-value results. Random assignment was performed to allocate the participants in the training set (n = 191) and validation set (n = 81), maintaining a ratio of 7:3. The flowchart detailing this process is depicted in Figure 1.

This study was approved by the ethics committee of the Panyu Hospital of Chinese Medicine approved this study, which waived the requirement for individual consent due to the use of retrospective data.

### Clinical variables

Previous studies have identified weight and age as significant risk factors for osteoporosis. Given their practical accessibility in clinical settings, we gathered age and weight as clinical factors for the participants who underwent DXA.

### Imaging feature extraction

Lateral lumbosacral radiographs from the included cases were collected and stored in Dicom format. Vertebral regions of interest (ROI) were manually delineated on the lumbar lateral radiographs by two spine surgeons (each with 5 years of experience and blinded to the osteoporosis status of the X-rays) using a 3D Slicer (version No.5.0.3). Subsequently, the accuracy of the ROIs was verified by a deputy chief physician with 10 years of experience. Normalization of the images was performed before the outlining process to improve the convergence speed of the model. The L1-L4 vertebrae were outlined using the same labels, and these labels were input into the 3D Slicer Radiomics expansion package. This package facilitated the automated extraction of imageomics-related features, including first-order features, grayscale covariance matrix, grayscale tour length matrix, grayscale size-band matrix, and neighboring grayscale difference matrix among others, describing both the morphological and textural features of the cones. Finally, the intraclass correlation coefficient (ICC) of the extracted features was computed, and only features with ICC >0.75 were included in this study.

### Model development

To avoid over-fitting and reduce computational complexity, we implemented dimensionality reduction for the radiomic features. Utilizing least absolute shrinkage and selection operator (LASSO) regression, we identified radiomic features most closely associated with osteoporosis occurrence. Subsequently, Spearman’s correlation analysis was employed to eliminate highly correlated features, resulting in the inclusion of 12 radiomic features for model development. The radiomics model consisted of a product combination of the final retained features and their corresponding weighting factors, and a radiomic score (rad-score) was then calculated. Clinical risk factors for osteoporosis underwent univariate analysis, with factors exhibiting a significance level of P < 0.05 being integrated in the logistic regression model. Finally, factors with P < 0.05 in the logistic regression were included in the clinical model modeling. The clinical and radiomics model was built based in the identified clinical and radiomics characteristics. A column-line diagram representing the clinical and radiomics model was created.

### Validation of the model

The performance of the model in the identification of osteoporosis was evaluated using the receiver operating characteristic (ROC) curves, comparing the AUC across the training and test sets for various models. Calibration curves were employed to evaluate the goodness of fit of the column plots, and data from the validation set were used to assess the validity of the column plots. Decision curve analysis (DCA) was employed as a method to assess the usability and efficiency of radiomics models, visually displaying the “net benefit” of a model. Positive net reclassification improvement (NRI) values indicate that the model provides a net improvement in clinical decision-making for patients with osteoporosis.

### Statistical analysis

Statistical analyses were performed using the R software. Initially, the Kolmogorov-Smirnov test was employed to examine whether the texture feature parameters adhered to a normal distribution. For continuous variables, the significance of differences was assessed using independent samples t-test or univariate analysis, depending on the normality of the distribution. The Mann-Whitney U test was applied in cases where the features exhibited a non-normal distribution. Categorical variables were compared between groups using the Fisher exact test or chi-square test. A significance level of P < 0.05 was considered statistically significant.

LASSO logistic regression analysis was performed using the “glmnet” extension in R. The filtered features were analyzed through Spearman correlation analysis and the final retained features exhibiting a correlation magnitude |R| >0.7 were excluded. The rad-score for each patient was computed as the sum of the products of the final retained features, based on the radiomic features and their corresponding coefficients. Finally, ROC curves, calibration curves, DCA curves, and column line plots were generated using the R language. The corresponding AUC was calculated, with a larger AUC indicating a more effective predictive performance of the model.

## Results

### Clinical outcomes

There were 191 patients in total in the training set, including 98 patients in the non-osteoporotic group, with a mean age of 66.02 ± 8.04 years and a mean weight of 55.74 ± 7.34 kg. Additionally, there were 93 patients in the osteoporotic group, with a mean age of 61.63 ± 8.53 years and a mean weight of 62.21 ± 9.77 kg, as outlined in Table 1.

**TABLE 1:** Clinical data for included patients.

	Osteoporosis (n = 98)	Non-osteoporotic (n = 93)	P-value
Age, y	66.02 ± 8.04	61.63 ± 8.53	0.000
Weight, kg	55.74 ± 7.34	62.21 ± 9.77	0.000

### Imaging histologic features

**FIGURE 2:**
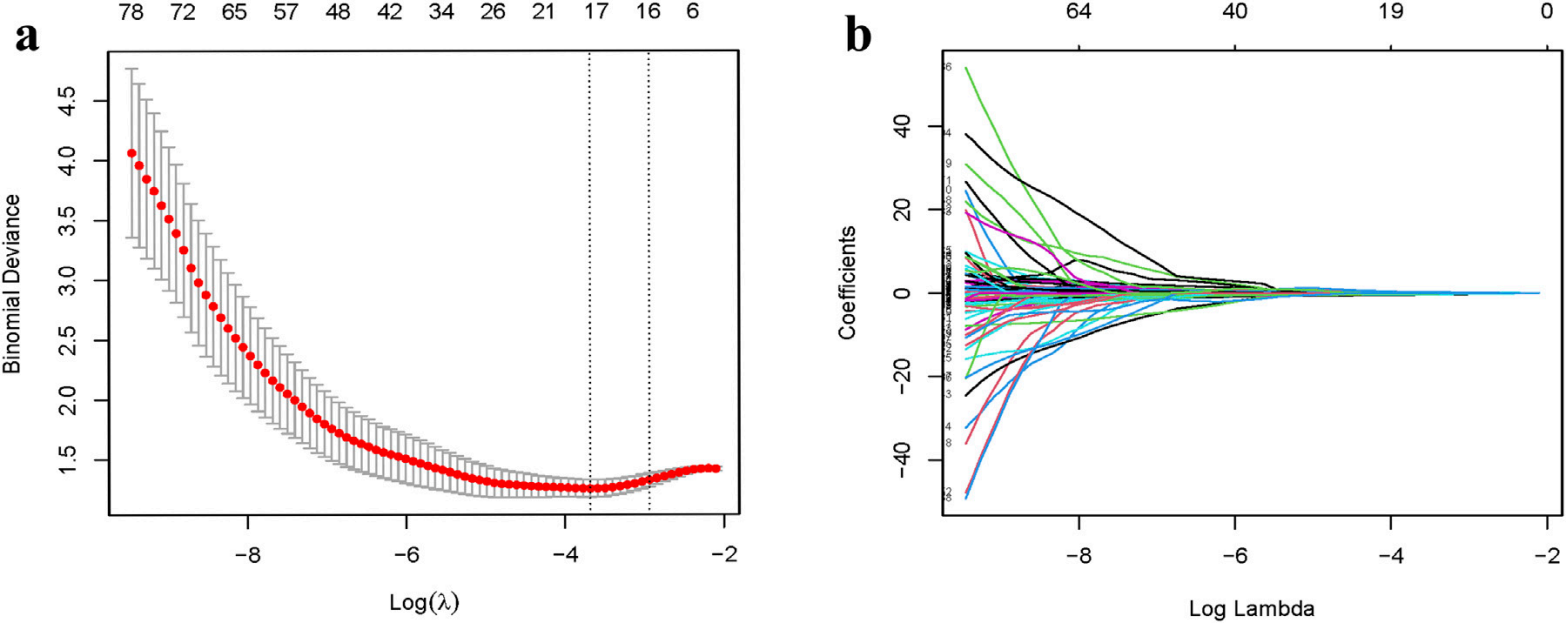
Lumbar X-ray radiomic feature selection based on LASSO regression **(a,b)**.

After employing R to classify the texture features, the optimal tunable parameter (λ = 0.0230) was identified, resulting in 17 image grouping features. Subsequently, through Spearman correlation analysis, features with high correlation (|R| > 0.7) were excluded, yielding 12 image grouping features (Figure 2). These features include wavelet-HLL_ firstorder_Mean, wavelet-HLL_firstorder_Skewness, wavelet-HHL_firstorder_Skewness, wavelet-HLL_glcm_ClusterShade, wavelet-HLL_glcm_ Idmn, wavelet-LHH_glszm_LowGrayLevelZoneEmphasis, wavelet-LHH_glszm_SizeZoneNonUniformity, wavelet-HLH_glszm_LargeAreaEmphasis, wavelet-HLH_glszm_SmallAreaEmphasis, wavelet-HHH_gldm_LargeDependenceHighGrayLevelEmphasis, wavelet-HHH_gldm_ SmallDependenceHighGrayLevelEmphasis, and wavelet-LLL_gldm_LargeDependenceLowGrayLevelEmphasis.

### Comparison of predictive models

**FIGURE 3:**
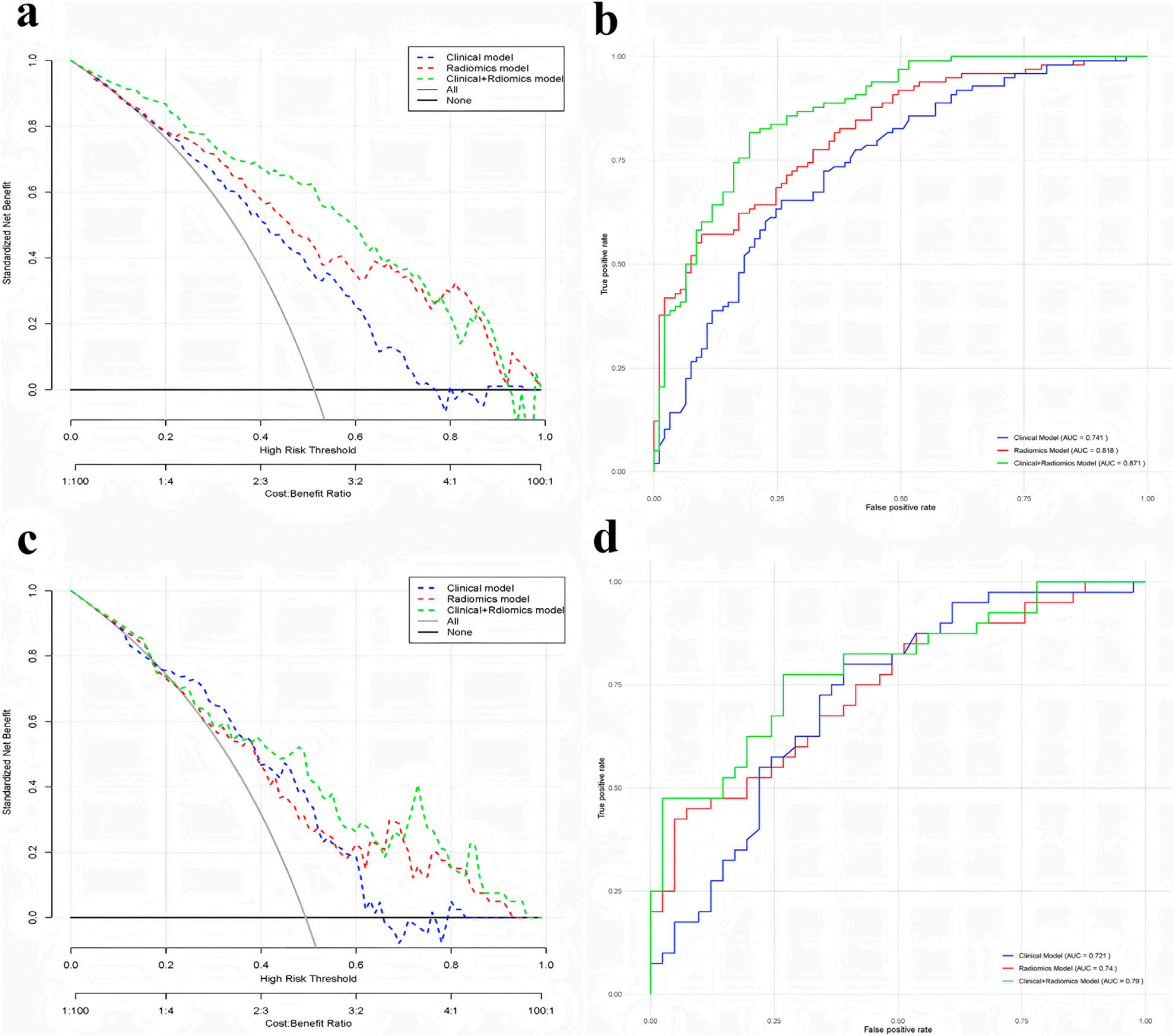
**(a)** Comparison of DCA curves for training-focused clinical model, radiomics model, and clinical + radiomics model; **(b)** Comparison of ROC curves for training-focused clinical model, radiomics model, and clinical + radiomics model; **(c)** Comparison of DCA curves for test-focused clinical model, radiomics model, and clinical + radiomics model; and **(d)** Comparison of DCA curves for testing-focused clinical model and radiomics model, clinical + radiomics Model ROC curve comparison.

**FIGURE 4:**
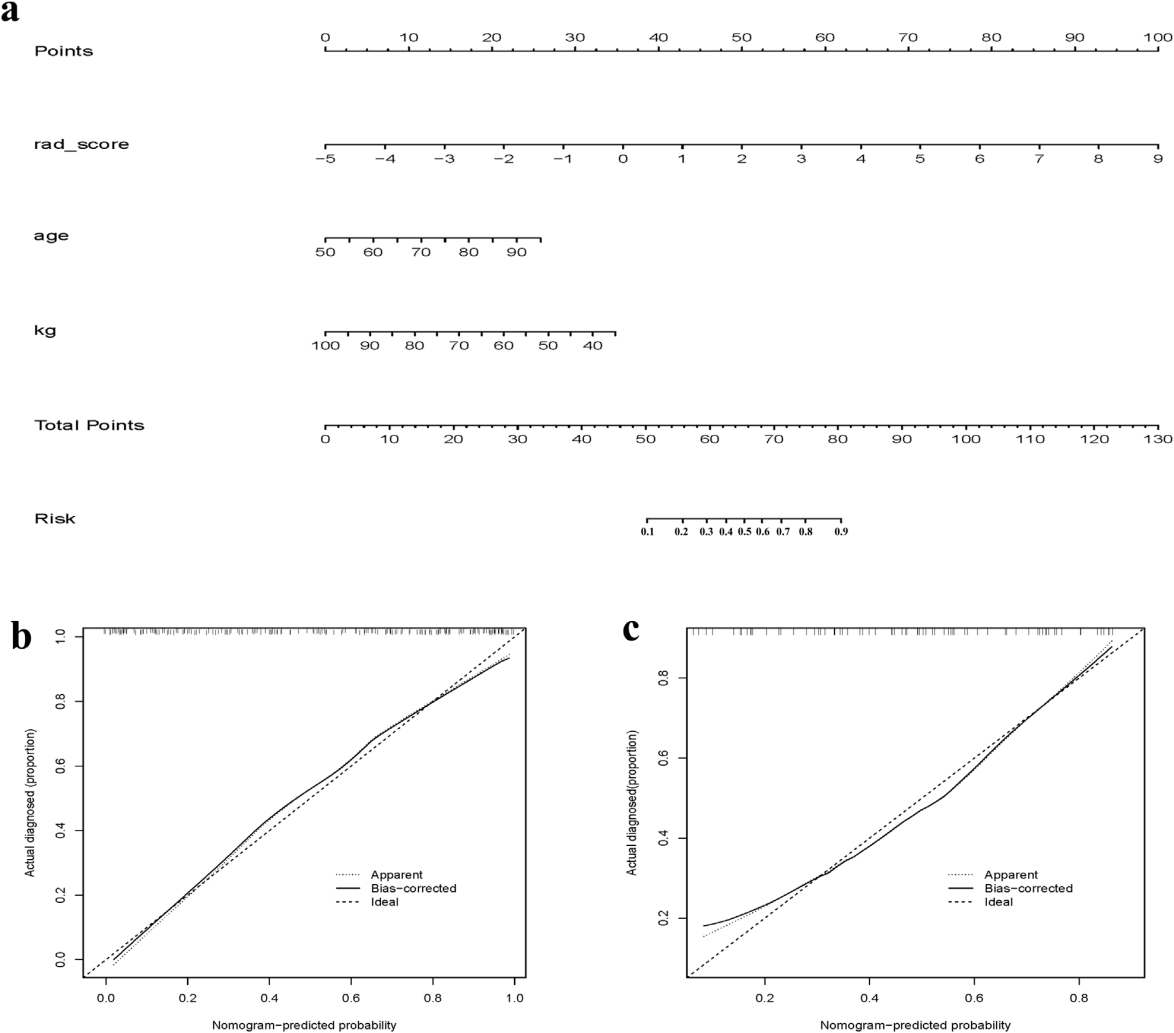
**(a)** Clinical + Radiomics column-line diagram clinical prediction model; **(b)** Clinical + Radiomics training set calibration curve; **(c)** Clinical + Radiomics test set calibration curve.

Both the radiomics modeling group and the radiomics plus clinical modeling group demonstrated superior results compared to the clinical modeling group (Figures 3, 4). In the radiomics modeling group, the training set and test set AUCs were 0.818 and 0.740, respectively, with a sensitivity and specificity of 57.1%, 90.3%, and 45.0%, 92.7%, respectively. The clinical modeling group achieved an AUC of 0.741 on the training set and 0.721 on the test set. The sensitivity and specificity were 65.3% and 74.2% for the training set, and 80.0% and 61.0% for the test set, respectively.The radiomics plus clinical modeling group demonstrated a training set AUC of 0.871, with a sensitivity of 81.6% and a specificity of 80.6%. In the test set, the AUC was 0.790, with a sensitivity of 77.5% and a specificity of 73.2% (Table 2).

**TABLE 2:** Comparison of the prediction results of the three models.

	Model	Sensitivity	Specificity	ROC	95% CI
Training set	Radiomics	57.10%	90.30%	0.818	0.763∼0.830
	Clinical	65.30%	74.20%	0.741	0.729∼0.766
	Radiomics + Clinical	81.60%	80.60%	0.871	0.843∼0.891
Test set	Radiomics	45.00%	92.70%	0.74	0.690∼0.776
	Clinical	80.00%	61.00%	0.721	0.709∼0.752
	Radiomics + Clinical	77.50%	73.20%	0.79	0.772∼0.803

## Discussion

Osteoporosis is a prevalent and extensively studied condition characterized by a weakening of bone structure and a reduction in bone density, often resulting in fragile fractures, particularly in areas such as the hip, spine, humerus, and wrist (Clynes et al., 2020). As a bone-related condition, early detection and appropriate management of osteoporosis are crucial for mitigating or preventing complications such as fractures (Viganò et al., 2023). Some studies have developed and validated radiographic characterization models employing MR and CT for pre-surgical detection of osteoporosis, particularly in lumbosacral surgeries. Radiological techniques offer valuable information that can aid in surgical decision-making without additional medical costs or radiation exposure (Cai et al., 2023; Jiang et al., 2022; He et al., 2021). Lumbosacral X-rays, as an economical and widely accessible screening modality, serve as a guiding tool for clinical decisions related to detection, medication, and follow-up for individuals at high risk for osteoporosis and fractures. Radiomic-based approaches have the potential utility and scalability to enhance osteoporosis risk prediction as an adjunct procedure to routine x-ray scans in clinical practice, including previously stored images.

In this study, we developed a clinical prediction model for osteoporosis using lateral lumbar X-ray images of the L1-L4 vertebrae from patients without vertebral fractures at our institution. The model employs radiologic features extracted from X-ray images of the L1-L4 vertebrae, allowing for the diagnosis of osteoporosis based on a patient’s lumbosacral X-rays. This image processing method is easy to implement and cost-effective, without introducing additional radiation hazards. Given the limited availability of DXA tests in primary hospitals, accurate diagnosis of osteoporosis can be challenging. Our clinical prediction model addresses this limitation by providing a means to identify the risk of osteoporosis through routine spinal X-rays. This approach offers a practical solution to diagnostic challenges in primary hospitals, contributing to resource optimization.

The development of osteoporosis is influenced by various factors, including sex, age, body mass index, and the use of certain medications (Khalifa et al., 2023), with a higher prevalence generally observed in postmenopausal women. The predictive model in this study was developed by incorporating easily accessible clinical factors, namely, age and body weight, in conjunction with radiologic features extracted from lumbosacral X-rays. The predictive model was categorized into three main groups: the radiomics model group, the clinical model group, and the radiomics plus clinical model group. Comparative assessments of their clinical performance were conducted by evaluating the AUC of the three model groups. It was found that the ROC of the radiomics plus clinical model group reached 0.871, with a sensitivity of 81.6% and a specificity of 80.6%, surpassing both the radiomics and clinical model groups. Based on the imaging features and clinical parameters of lumbosacral X-rays, and by combining the AUCs and columnar plots calculated in the best radiomics model group, it was found that the X-ray radiomics features could predict whether a patient had osteoporosis or not. Radiomics is the emerging principle of comprehensive and automated quantification of radiographic phenotypes using data characterization algorithms (E et al., 2008; Yao et al., 2022).

This study has several limitations that warrant consideration. First, the single-center retrospective design and relatively homogeneous study population may introduce selection bias despite statistical safeguards. Additionally, the relatively small sample size may affect the robustness and generalizability of our model, highlighting the need for external validation in larger, more diverse cohorts. Second, the mean age of participants was 66 years, which is relatively young compared to typical osteoporosis screening populations, so further investigation is needed to assess the model’s applicability in older individuals, particularly those over 70 or with multiple risk factors. Finally, the model was developed based on lateral lumbar X-rays (L1–L4), limiting its direct applicability to other skeletal sites such as the hip or forearm, or to patients with vertebral fractures. Moreover, incomplete documentation of comorbidities (e.g., diabetes, glucocorticoid use) may constrain the model’s predictive accuracy.

Although our model shows potential as a cost-effective alternative to DXA, successful clinical implementation will require validation of automated ROI delineation tools and standardized clinician training protocols. Our findings could be particularly valuable in regions or healthcare settings where DXA is unavailable or underutilized. Given that lumbosacral radiographs are frequently obtained for other clinical reasons, combining these images with radiomics-based analysis may offer an accessible and opportunistic screening strategy. Overall, this proof-of-concept study lays important groundwork for future multi-center prospective validations and adaptation to diverse populations and skeletal sites, ultimately aiming to enhance osteoporosis detection through affordable imaging modalities.

## Conclusion

In summary, the clinical prediction model developed in this study, integrating clinical and radiomics from lumbosacral X-rays, offers a generalizable solution that improves the diagnostic capabilities of primary hospitals in identifying osteoporosis. This further contributes to the reduction of social burdens associated with osteoporosis complications.

## Data Availability

The original contributions presented in the study are included in the article/supplementary material, further inquiries can be directed to the corresponding author.
